# Genetic variants of CTLA4 are associated with clinical outcome of patients with multiple myeloma

**DOI:** 10.3389/fimmu.2023.1158105

**Published:** 2023-04-12

**Authors:** Yolanda Gonzalez-Montes, Rocío Rodriguez-Romanos, Alicia Villavicencio, Gemma Osca-Gelis, Marta González-Bártulos, Francesca Llopis, Victòria Clapes, Albert Oriol, Anna Sureda, Lourdes Escoda, Josep Sarrà, Ana Garzó, Natàlia Lloveras, Isabel Díez, Isabel Granada, David Gallardo

**Affiliations:** ^1^ Hematology Department, Institut Català d’Oncologia, Hospital Dr. Josep Trueta, Institut d’Investigació Biomèdica de Girona (IDIBGI), Josep Carreras Research Institute, Girona, Universitat de Girona, Girona, Spain; ^2^ Girona Cancer Registry, Oncology Coordination Plan, Catalan Institute of Oncology (RTH) ICO-ICS, Centre CIBER of Epidemiology and Public Health (CIBERESP), Girona, Spain; ^3^ Clinical Hematology Department, Institut Català d’Oncologia, L’Hospitalet, IDIBELL, Universitat de Barcelona, Hospitalet de LLobregat, Spain; ^4^ Hematology Department, Institut Català d’Oncologia, Hospital Germans Trias i Pujol, Josep Carreras Research Institute, Badalona, Barcelona, Spain; ^5^ Hematology Department, Institut Català d’Oncologia, Hospital Joan XXIII, Universitat Rovira i Virgili (URV), Tarragona, Spain

**Keywords:** CTLA4 polymorphisms, multiple myeloma, immune checkpoint, bone marrow microenvironment, cytogenetics and molecular genetics

## Abstract

Immune dysfunction in patients with multiple myeloma (MM) affects both the innate and adaptive immune system. Molecules involved in the immune checkpoint pathways are essential to determine the ability of cancer cells to escape from the immune system surveillance. However, few data are available concerning the role of these molecules in predicting the kinetics of progression of MM. We retrospectively analysed polymorphisms of CTLA4 (rs231775 and rs733618), BTLA (rs9288953), CD28 (rs3116496), PD-1 (rs36084323 and rs11568821) and LAG-3 (rs870849) genes in 239 patients with newly diagnosed MM. Patients with a CTLA4 rs231775 AA/AG genotype showed a median progression-free survival (PFS) significantly lower than those with GG genotype (32.3 months versus 96.8 months respectively; p: 0.008). The 5-year PFS rate was 25% for patients with grouped AA and AG genotype vs 55.4% for patients with GG genotype. Multivariate analysis confirmed the CTLA4 rs231775 genotype as an independent risk factor for PFS (Hazard Ratio (HR): 2.05; 95% CI: 1.0-6.2; p: 0.047). Our results suggest that the CTLA4 genotype may identify patients with earlier progression of MM. This polymorphism could potentially be used as a prognostic biomarker.

## Introduction

Multiple myeloma (MM) is a neoplastic plasma-cell disorder that is characterized by clonal proliferation of malignant plasma cells in the bone marrow, monoclonal protein in the blood or urine and associated organ dysfunction. MM develops from a premalignant condition monoclonal gammopathy of undetermined significance (MGUS), progressing to smoldering multiple myeloma and active MM. Progressive immune impairment is a feature of this MM evolution, allowing neoplastic plasma cells to escape from immune surveillance, promoting disease growth and resistance to therapy (1, 2). Survival of patients with MM has improved during the past decade with the introduction of immunomodulatory drugs, proteasome inhibitors and monoclonal antibodies. Unfortunately, it remains an incurable disease characterized by recurring relapses due to residual, drug-resistant, myeloma cells that survive to the treatment (3).

The adaptive immune system can recognize and attack malignant cells. It is generally considered that T cells specific to tumour antigens play a crucial role in cancer elimination. T cell activation is initiated through tumour antigen recognition by the T cell receptor (TCR) and is regulated by a balance between activating and inhibitory intracellular signals. These signals are initiated by engagement of co-stimulatory and co-inhibitory receptors with their cognate ligands. The balance between positive and negative co-signals determines the functionality of T cells during immunity and tolerance, preventing the appearance of autoimmune phenomena. The ability of cancer cells to evade anti-tumour T-cell activity has been recognised as an important mechanism of cancer progression. Malignant cells can enhance the expression of inhibitory immune checkpoint molecules to avoid immune recognition and elimination (4).

The pathogenesis of the immune dysregulation in MM is associated with impaired cellular immunity including profound T cell alterations with loss of effector function and increased number of immunosuppressive regulatory T cells (Treg) in the bone marrow (2). A major role in this development of the immunosuppressive state in MM patients has been attributed to an increased expression of immune checkpoint molecules that negatively regulate T-cell function, such as PDCD1, CTLA4, BTLA and T-cell immunoglobulin and ITIM domains (TIGIT) on T cells (5–7).

Genetic variants of immune checkpoint molecules have been identified as risk factors for cancer development (8). Several studies have shown a relationship between genetic polymorphisms in co-stimulatory/inhibitory molecules and susceptibility to the development of hematologic malignancies, such as non-Hodgkin lymphomas (9, 10), chronic lymphocytic leukemia (11, 12), and MM (13–17). In addition, some studies have described the correlation between CTLA4 and PDCD1 genotypes and the clinical outcome after allogeneic stem cell transplantation (18, 19) or with the risk of acute myeloid leukemia progression after achieving complete remission (20).

However, few data are available concerning the clinical impact of genetic polymorphisms in co-stimulatory/inhibitory molecules on clinical outcome in patients with MM. In our study we intend to evaluate whether the presence of genetic variations of these checkpoint molecules is associated with an increased risk of progression in patients with MM.

## Patients and methods

We retrospectively analysed 239 patients with newly diagnosed MM who were eligible for first-line treatment and followed at the Catalan Institute of Oncology centers between 1995 and 2018. DNA was obtained from peripheral blood or bone marrow samples at different stages of the disease. Biological samples and clinical data were processed following standard operating procedures and approved by the Ethics and Scientific Committees. All patients signed an informed consent and the study met with the recommendations of the Helsinki declaration. Samples and data from patients included in this study were provided by the IDIBGI Biobank (Biobanc IDIBGI, B.0000872), integrated in the Spanish National Biobanks Network and they were processed following standard operating procedures with the appropriate approval of the Ethics and Scientific Committees. Clinical characteristics of patients and first-line treatments are summarized in **Table 1**.

**Table 1:** Clinical characteristics the studied cohort.

Characteristics (N = 239)	Total
	%^a^
Age (years)	
Median (range)	68 (61-76)
Sex	
Men	56.5
Women	43.5
Type of Monoclonal protein	
IgG	53.1
IgA	27.2
Light chains	13.4
Others	6.3
History of MGUS	8.1
ISS stage	
I-II	70.4
III	29.6
LDH	
High	8.5
Normal	91.5
Kidney failure ^ł^	
No	77.6
Yes	22.4
PBSCT	
Yes	40.9
No	59.1
First Line Therapy	
Alkylating agents	20.5
Proteasome inhibitors	53.1
Immunomodulatory drugs	5.4
Proteosome Inhibitors	20.5
Immunomodulators	20.5
Daratumumab monotherapy	0.4

^a^ Except where specified.

MGUS, monoclonal gammopathy of undetermined significance; **^ł^** Kidney failure: creatinine ≥ 2 mg/dl; PBSCT, Progenitor cell transplantation. LDH, lactate dehydrogenase.

In addition, a second cohort of 62 patients with newly diagnosis of smouldering MM was specifically analysed to correlate the genotype in the studied polymorphisms with the time to progression to symptomatic MM.

### Genotype analysis

DNA was extracted from 200µl of whole blood using a QIAamp DNA Blood Mini Kit (Qiagen, GmbH, Hilden, Germany) according to the manufacturer’s instructions and stored at -80°C until use.

We analysed polymorphisms of CTLA4 (rs231775 and rs733618), BTLA (rs9288953), CD28 (rs3116496), PDCD1 (rs36084323 and rs11568821) and LAG3 (rs870849) genes. The genotype for these polymorphisms was determined *via* allelic discrimination plots on Applied Biosystems™ QuantStudio™ 7 Flex Real-Time PCR System by using TaqMan^®^ SNP Genotyping Assays real time PCR according to the manufacturer’s instruction.

### Statistical analyses

Allele frequencies and genotypes were formulated by direct counting. Hardy-Weinberg equation (p^2^ + 2pq + q^2^ = 1) was used to measure whether the observed genotype frequencies in the studied population differ from the predicted frequencies. Homogeneity between genotype groups was evaluated using the chi-square test or Fisher’s exact test for qualitative variables and Student’s test for continuous variables. Kaplan-Meier curves were derived to determine overall survival (OS) and progression free survival (PFS) and curves were compared using the log-rank test. A two-sided p value of 0.05 or lower was considered to be statistically significant. Multivariate analysis was performed using the Cox regression model. All the variables with a p value at or below 0.2 in the univariate analysis were included in the multivariate analysis.

## Results

### CTLA4, BTLA, CD28, PDCD1 and LAG3 genotype distribution.

**Table 2** shows the genotypes distribution for each analysed polymorphism. The genotype frequencies were comparable to the previously described in Caucasian population.

**Table 2:** Frequencies of genetics polymorphisms in the analysed patients.

Genes	Genotype	(%)	
		MM	smouldering MM
CTLA4 rs231775	AA	46.6	56.4
	GG	9.5	5.1
	AG	44.0	38.5
CTLA4 rs733618	CC	0.0	0.0
	TT	92.5	95.2
	CT	7.5	4.8
BTLA rs9288953	CC	62.2	63.2
	TT	5.2	7.9
	CT	32.6	28.9
PDCD1 rs36084323	CC	93.2	94.6
	TT	0.0	2.7
	CT	6.8	2.7
PDCD1 rs11568821	CC	82.3	73.0
	TT	1.3	0.0
	CT	16.4	27.0
LAG3 rs870849	CC	35.7	35.1
	TT	49.3	43.2
	CT	14.9	21.6

### Homogeneity between genotype groups

The comparison of clinical prognostic factors at diagnosis between genetic groups for each polymorphism showed a balanced distribution for age, sex, type of monoclonal protein, former history of monoclonal gammopathy of unknown significance (MGUS), International Staging System (ISS), cytogenetics, serum LDH levels or kidney failure. Moreover, the proportion of patients receiving an autologous peripheral blood stem cell transplant (PBSCT) was also comparable within genetic groups. **Tables S1**, **S2** show the comparison of clinical characteristics of the symptomatic MM patients according to the genetic groups according to their BTLA (rs9288953), CD28 (rs3116496), PDCD1 (rs36084323 and rs11568821), LAG3 (rs870849) and CTLA4 (rs231775 and rs733618) genotypes.

### Correlation between immune checkpoint molecules genotype and progression to symptomatic myeloma

When analysing the cohort of 62 patients with newly diagnosed smouldering MM, we did not find any correlation between the analysed genotypes and the time to receive a first line of therapy. In this cohort, the median the time to receive therapy was 22.8 months for patients with CTLA4 rs231775 GG genotype versus 102 months for those patients with the AA+AG genotype (p: 0.7). Similar results were obtained when analyzing the impact of the CTLA4 rs733618 (CC+CT: 174.5 months vs. TT: 65.2 months; p: 0.4), BTLA rs9288953 (CC+CT: 65.2 months vs. TT 135.1 months; p: 0.6), CD28 rs3116496 (CC+CT: 61.6 months vs. TT: 135.1 months; p: 0.7), PDCD1 rs36084323 (CC+CT: 102 months vs. TT: not reached; p: 0.6) and LAG3 rs870849 (TT+CT: 61.6 months vs. CC: 122.6 months; p: 0.3) polymorphisms.

### Immune checkpoint molecules genotype and clinical outcome after treatment

We did not find any significant correlation between polymorphisms of BTLA (rs9288953), CD28 (rs3116496), PDCD1 (rs36084323 and rs11568821), LAG3 (rs870849) or CTLA4 (rs733618) and overall survival or progression-free survival (PFS) (**Table 3**).

**Table 3:** Overall and progression-free survival of multiple myeloma patients according to immune checkpoint genotypes.

Genes	OS (95% CI)			PFS (95% CI)		
	5-year	10-year	*P*	5-year	10-year	*P*
CTLA4 rs231775						
AA+AG	65.8 (59.6-73.0)	37.0 (29.1-47.2)	0.9	25.0 (19.0-33.0)	10.5 (5.2-21.1)	0.008
GG	69.2 (51.0-93.9)	43.2 (20.7-90.2)		55.4 (34.3-89.3)	36.9 (14.5-93.7)	
CTLA4 rs733618						
CC+CT	72.9 (53.1-100.0)	39.1 (17.5-87.0)	0.9	42.6 (21.4-84.6)	–	
TT	65.4 (59.1-72.4)	38.1 (30.3-48.0)		27.6 (21.4-35.5)	13.6 (7.8-23.6)	
BTLA rs9288953						
CC+CT	65.9 (59.5-72.9)	36.4 (28.4-46.7)	0.4	29.2 (22.9-37.3)	15.1 (8.9-25.5)	1.0
TT	79.5 (57.7-100)	56.8 (32.2-100)		21.2 (6.3-71.6)	10.6 (1.7-67.1)	
CD28 rs3116496						
CC+CT	71.4 (64.3-79.2)	39.4 (30.6-50.7)	0.2	30.1 (20.3-44.4)	14.2 (6.9-28.9)	0.3
TT	56.8 (45.2-71.5)	37.5 (21.3-66.0)		28-1 (20.7-38-2)	14.1 (5.6-35.2)	
PDCD1 rs36084323						
CC+CT	66.4 (60.2-73.1)	38.0 (30.3-47.7)	0.1	28.5 (22.4-36.3)	13.7 (7.9-23.7)	–
TT	100.0 (100.0-100.0)	100.0 (100.0-100.0)		–	–	
PDCD1 rs11568821						
CC+CT	66.1 (59.9-73.0)	38.0 (30.3-47.6)	0.4	27.9 (21.8-35.8)	13.6 (7.8-23.5)	0.8
TT	100.0 (100.0-100.0)	100.0 (100.0-100.0)		–	–	
LAG3 rs870849						
TT+CT	67.4 (59.7-76.1)	38.9 (29.3-51.8)	0.2	26.8 (19.4-37.0)	11.5 (5.3-24.7)	0.3
CC	60.1 (49.6 -72.9)	31.3 (20.1-48.6)		29.9 (20.0-44.8)	11.9 (2.7-52.2)	

OS, overall survival; PFS, progression-free survival; 95% CI, 95% confidence interval.

**Figure 1:**
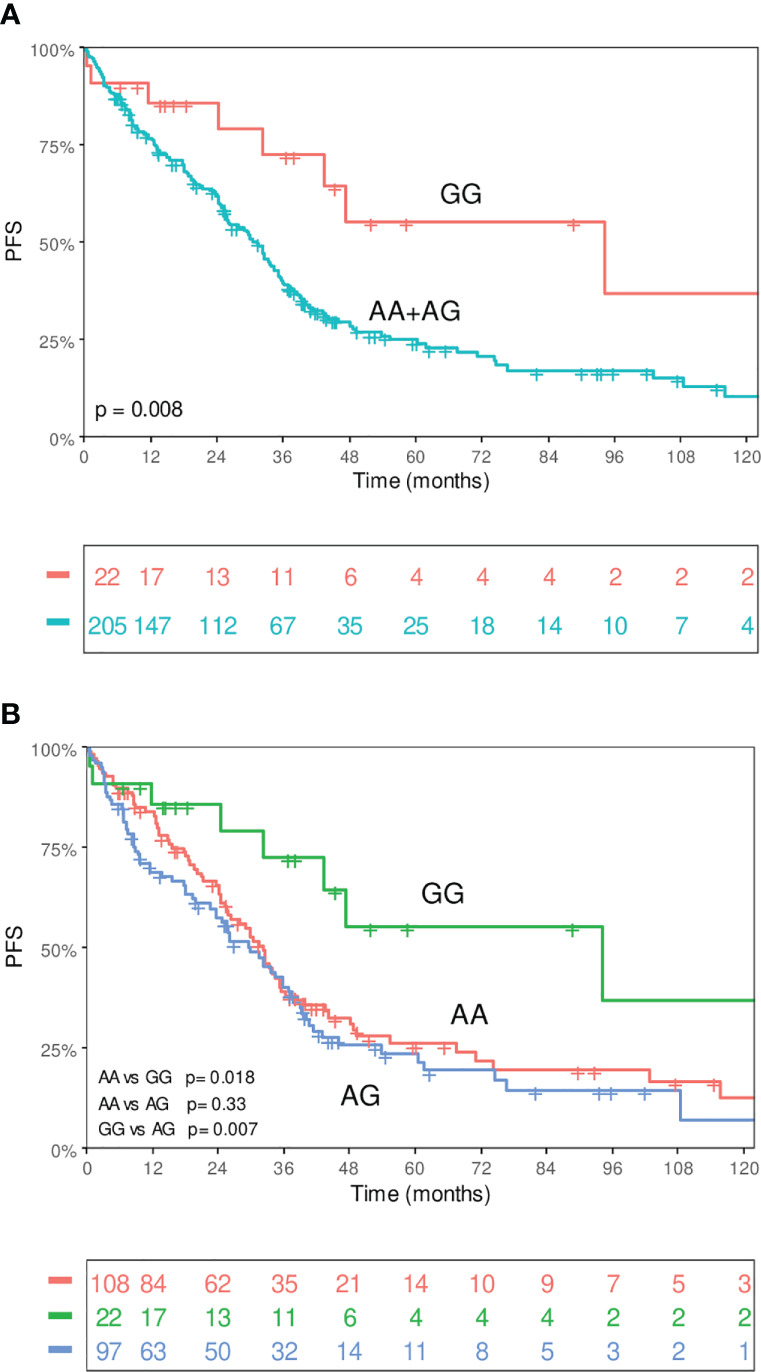
Kaplan-Meier curves of PFS according to CTLA4 rs2311775. **(A)** grouped phenotypes and **(B)** independent genotypes.

However, the analysis according to the CTLA4 rs231775 polymorphism revealed that the presence of the AA+AG genotype was associated with a median PFS significantly shorter than the GG genotype: 32.3 months (95% confidence interval (95% CI): 26.4 – 36.1) and 96.8 months (95% CI: 44.5 – not reached) respectively (p: 0.008) (**Figure 1**). Moreover, the median time to subsequent myeloma therapy was significantly lower for patients with CTLA4 rs231775 AA and AG genotype (36.3 months; 95% CI: 33.5-43) than in the patients with GG genotype (not reached; 95% CI 44.6-not reached) (p: 0.01).

The 5-year PFS rate was 25% for patients with grouped AA and AG genotype vs 55.4% for patients with GG genotype. Male gender (30.8 vs 36.4 months; p: 0.03), age ≥ 69 years (30.2 vs 36.8 months; p: 0.02), high-risk cytogenetics (18.6 vs 33.5 months; p: 0.01), high ISS (24.2 vs 38.5 months; p: 0.02) and not receive a hematopoietic stem cell transplantation (39.6 vs 26.6 months; p < 0.001) were also risk factors for lower PFS.

In the multivariate analysis, the CTLA4 rs231775 polymorphism remained as an independent risk factor for PFS (Hazard Ratio (HR): 2.05; 95% CI: 1.0-6.2; p: 0.047). **Table 4** shows the results of the multivariate analysis.

**Table 4:** Univariate and multivariate analysis of OS and PFS in multiple myeloma patients.

	Univariate analysis				Multivariate analysis			
	OS		PFS		OS		PFS	
	HR (95% CI)	*P*	HR (95% CI)	*P*	HR (95% CI)	*P*	HR (95% CI)	*P*
Sex								
Men	1.5 (1.0-2.1)	0.04	1.4 (1.0-2.0)	0.031	1.7 (1.1-2.6)	0.013	1.5 (1.0-2.2)	0.027
Age group								
> 69 years	2.3(1.5-3.3)	<0.001	1.5 (1.1-2.0)	0.020	0.9 (0.5-1.6)	0.741	0.6 (0.3-1.0)	0.050
ISS stage								
III	1.7 (1.1-2.7)	0.02	1.5 (1.1-2.3)	0.021	1.8 (1.1-2.8)	0.022	1.4 (1.0-2.1)	0.071
HSCT								
Non-HSCT	2.5 (1.6-3.9)	<0.001	1.9 (1.3-2.6)	<0.001	2.9 (1.6-5.5)	<0.001	2.9 (1.6-5.0)	<0.001
Genotype CTLA4 rs231775								
AA+AG	1.0 (0.5-2.0)	0.924	2.6 (1.2-5.3)	0.008	0.8 (0.4-1.9)	0.684	2.5 (1.0-6.2)	0.047

HSCT, hematopoietic stem cell transplantation; OS, overall survival; PFS, progression-free survival; HR, hazard ratio; 95% CI: 95% confidence interval.

**Figure 2:**
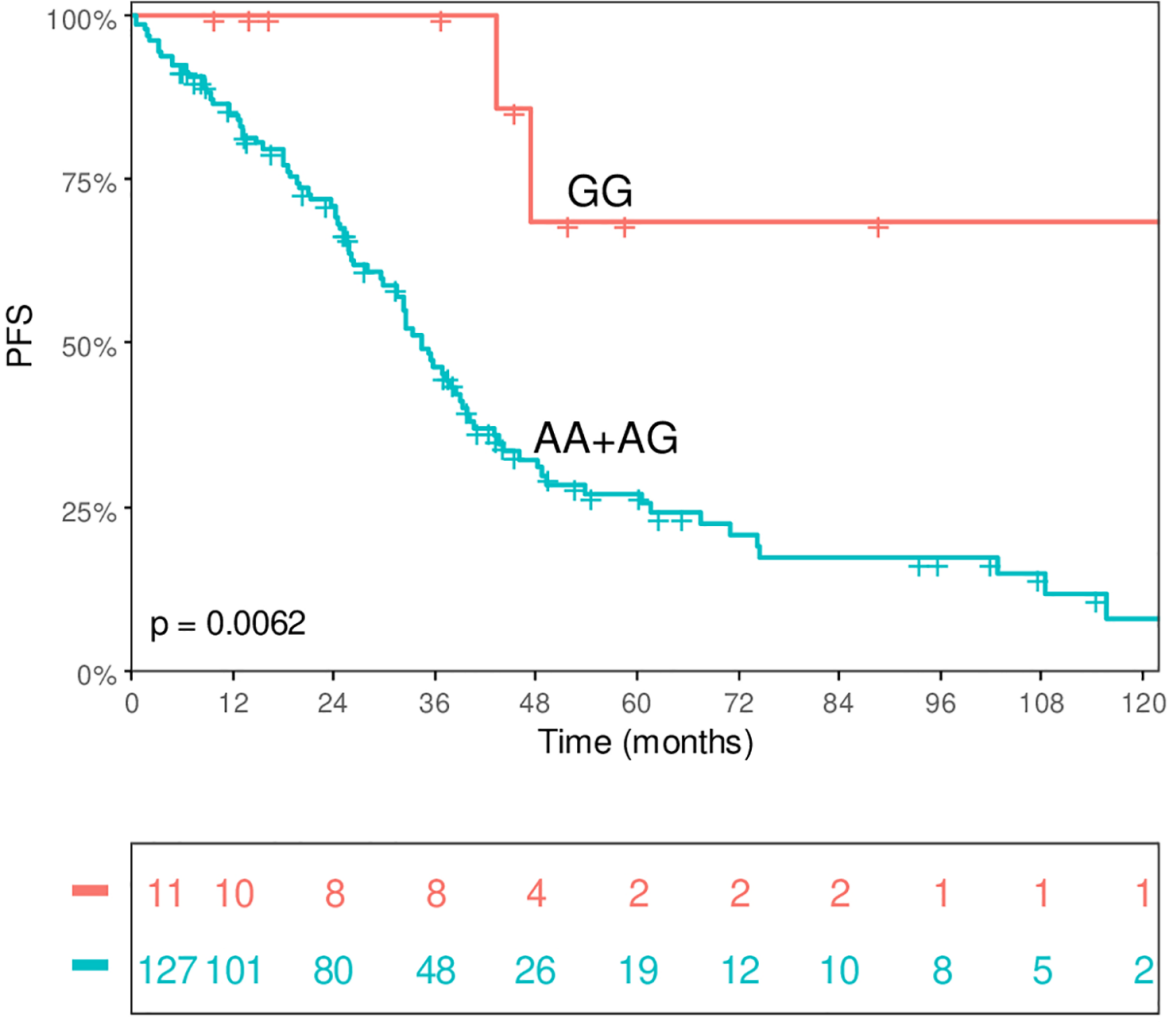
PFS for patients with ISS I-II according to CTLA4 rs2311775 genotype.

When performing subgroup analysis of patients with ISS I and ISS II, we detected more evident differences in PFS depending on the CTLA4 genotype. Interestingly, we observed that patients with GG genotype showed a much longer PFS (median not reached) than patients with AA+AG genotype, who had a median PFS of 34.3 months (95%CI: 31.4 – 39.8; p: 0.0062) (**Figure 2**).

**Figure 3:**
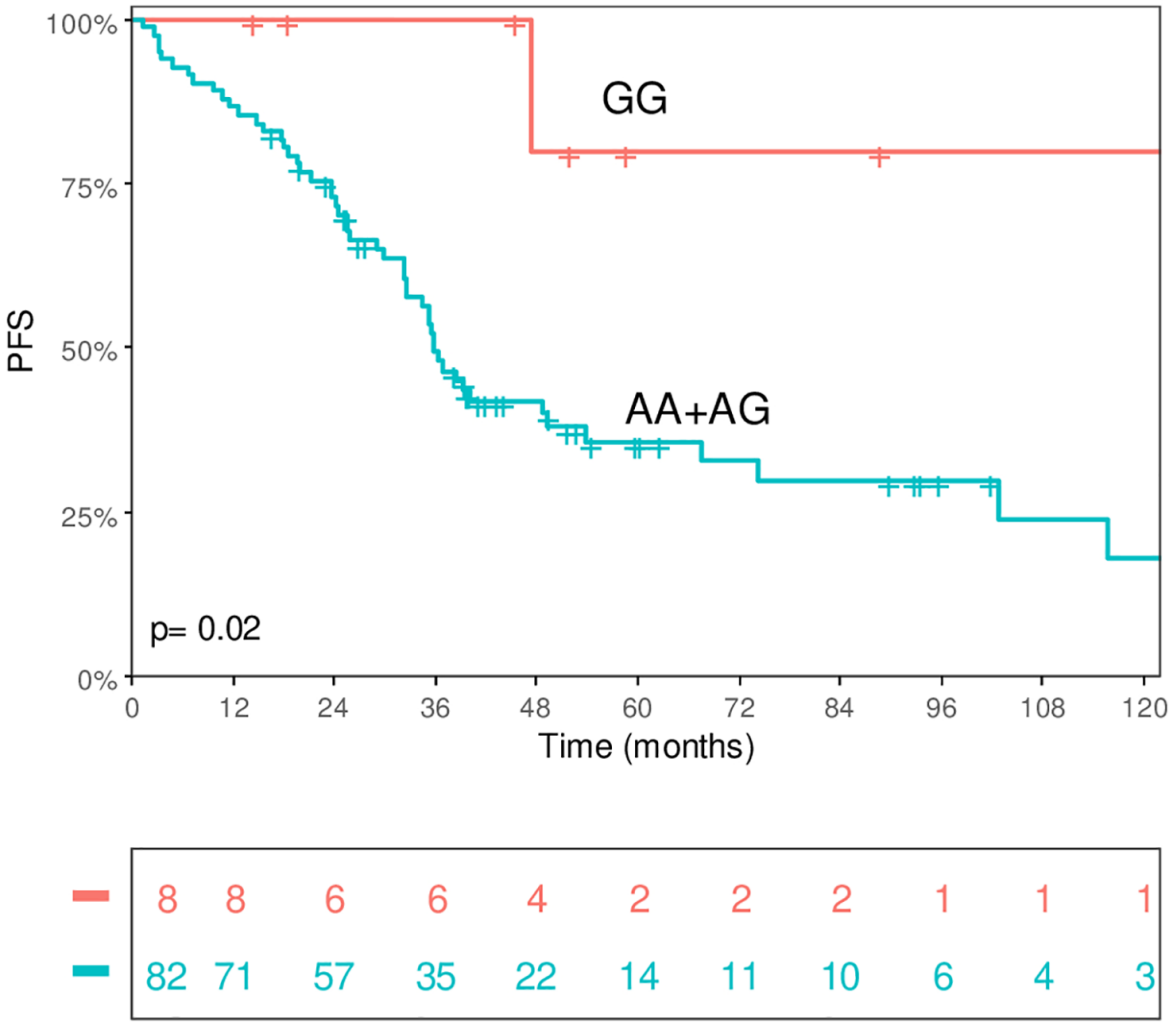
PFS for patients for patients receiving autologous stem cell transplant according to CTLA4 rs2311775 genotype.

In patients that received an autologous peripheral blood stem cell transplant (PBSCT) the AA+AG genotype was associated with a median PFS significantly lower than those with GG genotype: 35.9 months (95% CI: 32.6 – 53.7) and not reached respectively (p 0.02) (**Figure 3**). In contrast, no significant differences were observed in patients who did not receive auto-SCT.

## Discussion

Tumor progression depends upon the acquisition of traits that allow cancer cells to evade immune surveillance and suppress an effective immune response. The immune dysfunction and neoplastic evasion in MM is facilitated by multiple cytokine and cellular signaling pathways, which decrease immune effector cell function and determine a suppressive bone marrow microenvironment (2). This impaired cellular immunity is mediated by an increased number and functional impairment of dendritic cells (21), altered circulating CD4/CD8 ratio (22), decreased Th1/Th2 ratio (23), increased number of Treg (24), lower level of naïve and transitional B cells subsets in the bone marrow (25), increased number of myeloid derived suppressor cells (MDSC) in the blood and in the bone marrow (26), and enhanced expression of inhibitory ligands such as programmed cell death ligand 1 (PDL-1) by myeloma and bone marrow microenvironment cells, together with an increased expression of PDCD1 and CTLA4 on tumor infiltrating T cells (27, 28). The immunosuppressive state in MM determines important clinical implications, there is increasing evidence that the resistance to bispecific T cell engagers (TCEs) is closely related to immunosuppressive tumor microenvironment (29). Recently, Friedrich et al. (30) has shown that a high proportion of clonal exhausted-like CD8+ T cell clones in the bone marrow of TCE-receiving patients predicts response failure in MM.

We have detected an association between the CTLA4 rs231775 genotype and PFS in patients with MM. The human CTLA4 gene is located on chromosome 2q33. Several single nucleotide polymorphisms (SNPs) have been identified within this gene, and some of them have been related with the ability of CTLA-4 to inhibit immune responses, playing an important role in the development of autoimmune diseases (31) or cancer (32). The rs231775 polymorphism is a non-synonymous SNP which affects exon 1, leading to an alanine-to-threonine amino acid substitution at codon 17 in the leader peptide (Thr17Ala). The association of genetic variants of CTLA4 have been described to be more frequent in MM patients compared with matched healthy controls. Zheng C et al. (13) showed that the CTLA4 microsatellite polymorphism might represent a susceptibility locus for MM and MGUS. Karabon et al. (14) found that CTLA-4c.49A>G[G], CT60[G], and Jo31[G] alleles were more frequently observed in MM patients than in healthy controls. However very little information is available about the role of genetic variants of CTLA-4 in determining progression kinetic of patients with MM.

Several studies have established that T cells from MM patients are able to recognize and eliminate malignant cells (33, 34) and it has recently been determined that T cell exhaustion and a suppressive bone marrow microenvironment are implicated in MM progression (7, 28). CD8+T cell exhaustion and immune checkpoint receptor expression, in both transplant and non-transplant settings, could have an active role for the disease progression (7, 35).

The immune-based therapies offer an increased potential for tumor cell control and may reverse the lack of responsiveness of T cells (36). However, the initial experience with immune checkpoint inhibitors in MM patients has shown limited efficacy, significant toxicity, and side effects. Preclinical data show a potential utility of PD-1/PD-L1 blockade in MM therapy, but early clinical trials have been discouraging (37). Pembrolizumab immunotherapy did not show any activity in MM, and its combination with IMiDs, lenalidomide, or pomalidomide in relapsed or refractory MM patients was associated with immune-related toxicities and mortality (38, 39). While PD-1 blockage has not demonstrated clinical benefits in MM patients, it is notable that some patients achieved long-term remissions after stopping pembrolizumab in clinical trials (40). As well, a phase 1 study of nivolumab in combination with ipilimumab for relapsed or refractory hematologic malignancies did not demonstrate favourable results in MM (41). Currently, TIGIT has emerged as an attractive target for MM immunotherapy. News studies have demonstrated that blocking TIGIT using monoclonal antibodies increased the effector function of MM patient CD8+ T cells and prolongs survival in preclinical MM models (6, 42). Therefore, predictive biomarkers are needed in daily practice to identify the patients who would obtain benefit from immunological checkpoint blockade therapies.

In our study CTLA4 rs733618 polymorphism was not associated with significant differences on PFS and OS, even when considering to the type of anti-myeloma therapy received, as opposed to the results of Xiao-Ying et al. (43). To explain this discrepancy, we must consider the differences in the genetic background between European and Asiatic population: the population of our study was mainly Caucasian, which rarely expresses the CTLA4 rs733618 CC genotype, whereas this allele is more prevalent in Chinese people.

In the present study, the rs231775 CTLA4 AA/AG genotype was associated with lower PFS compared with the GG genotype. Additionally, the analysis identified a subgroup of long-term survivors of myeloma for ISS stage I or II with GG genotype. This finding can be explained by the effect of the G allele and the related lower function of CTLA4 and the reduced inhibition of activated T-cells (44, 45), leading to a more intense T-lymphocyte activity and a stronger immune surveillance. These findings need to be confirmed in other cohorts, due to the low incidence of the rs231775 GG genotype.

There is increasing evidence that the clinical benefit of autologous stem cell transplantation (auto-SCT) is consequence of the cytoreduction with chemotherapy combined with immunological changes. Recent studies suggest that auto-SCT produces immunomodulatory effects including inflammatory cytokine production, immunogenic cell death, enhanced antigen presentations and microenvironment disruption (42, 46). These effects enhance the immune-mediated myeloma control and appears to restore an immune equilibrium. The expansion of T cell clones following autologous transplant, and early lymphocyte recovery has been associated with longer PFS suggesting that early immune reconstitution contributes to control of disease progression in MM (46, 47). Based upon these observations, the minimal residual disease state and lymphopenia after auto-SCT provide an excellent platform to promote the incorporation of immune-based therapies into post auto-SCT treatment regimens to induce or restore antitumor immunity. Consistent with this, our results suggest an association between the rs231775 CTLA4 genotype and PFS after auto-SCT. Despite no checkpoint inhibitor is currently approved in multiple myeloma, our results suggest that this treatment strategy may benefit a subset of multiple myeloma patients.

Several clinical trials have investigated the clinical benefits of checkpoint inhibitors a consolidation after stem cell transplantation in MM. Interim data of a phase I/II study of nivolumab with autologous-SCT in patients with suboptimal response to primary induction, described a 56% of improved response and an acceptable toxicity (48). In a phase 2 study, pembrolizumab in monotherapy was administered in patients who did not achieve a complete response to induction therapy. The study was terminated early after failing to meet its interim analysis endpoint to detect a 20% difference in rate of complete response conversion at the end of treatment (49). Combined checkpoint inhibitors with nivolumab and ipilimumab was analysed in a Phase Ib-IIA study as consolidation after autologous-SCT in patients at high risk for post-transplant recurrence, SLP and SG 18 months post-SCT were 57.1% and 87% high-risk transplant-naïve MM, and 40% and 100% for MM relapsed within 3 years of first ASCT, the treatment was considered to have been well tolerated with no significant unexpected toxicity (50). The combination of antibodies against CTLA-4 and PD-1 was studied in a phase 1 trial to assess safety and tolerability of tremelimumab and durvalumab, administered with high dose chemotherapy and autologous stem cell transplant. FDA ordered to terminate the study due to safety signals in other studies investigating combination regimens comprising similar drugs. Because only 6 subjects were enrolled, no final statistical analysis plan was issued (51). Currently, interesting clinical trial are ongoing such as a pilot study phase 2 of lenalidomide alternating with ipilimumab post allogeneic and autologous SCT in treating patients with hematologic or lymphoid malignancies (NCT01919619). Also, a phase II study of targeting CD28 in multiple myeloma with abatacept (CTLA4-Ig) determine the therapeutic efficacy to overcome resistance to chemotherapy (NCT03457142).

Our results are encouraging and support the hypothesis that this CTLA4 polymorphism could be used as a biomarker to predict the outcome of MM and we suggest that genetic variants of CTLA-4 should be used as genetic marker to identify patients with high risk of progression, who may benefit of anti-CTLA-4-based therapy. Specific studies are needed to confirm this hypothesis.

Clinical trials are ongoing to evaluate strategies that may enhance immunity response, by directly promoting T cell activity against myeloma cells, including checkpoint inhibition, bispecific T-cell engagers and chimeric antigen receptor T cells. According to our results patients with AA/AG genotype of rs231775 CTLA4 may be ideal candidates for participation in such trials to analyse the clinical impact of selected combination immune-based therapies. Further studies with exploratory immunological endpoints are needed to establish the significance of immune gene variation for future clinical interventions for patients with MM.

## Data availability statement

The datasets presented in this study can be found in online repositories. The names of the repository/repositories and accession number(s) can be found in the article/**Supplementary Material**.

## Ethics statement

The studies involving human participants were reviewed and approved by Comitè d’Ètica d’investigació amb Medicaments CEIM Girona. The patients/participants provided their written informed consent to participate in this study.

## Author contributions

YG-M conceived and designed the study. RR-R, AV, GO, MG-B and FL participated in data collection; AV and GO participated in data analysis. DG participated in manuscript drafting. All authors revised the manuscript critically for important intellectual content, gave their final approval of the version to be published and agreed to be accountable for all aspects of the work in ensuring that questions related to the accuracy or integrity of any part of the work are appropriately investigated and resolved.
