# Supplementary material for: Genetic variants of CTLA4 are associated with clinical outcome of patients with multiple myeloma
Source: Front Immunol. 2023 Apr 12;14:1158105. doi: 10.3389/fimmu.2023.1158105 (PMC10143497; doi:10.3389/fimmu.2023.1158105)
Supplement: Supplementary file 1 [file Table_1.docx]

**Table S1.** Patients’ characteristics according to BTLA rs9288953, CD28 rs3116496, PDCD1 rs36084323, PDCD1 rs11568821 and LAG3 genotypes.

|  | **Total** |  | **BTLA rs9288953** | |  |  | **CD28 rs3116496** | |  |  | **PDCD1 rs36084323** | |  |  | **PDCD1 rs11568821** | |  |  | **LAG3 rs870849** | |  |  |
| --- | --- | --- | --- | --- | --- | --- | --- | --- | --- | --- | --- | --- | --- | --- | --- | --- | --- | --- | --- | --- | --- | --- |
|  | ***%a*** |  | **CC+CT** | **TT** | ***P*** |  | **TT+CT** | **CC** | ***P*** |  | **CC+CT** | **TT** | ***P*** |  | **CC+CT** | **TT** | ***P*** |  | **TT+CT** | **CC** | | ***P*** |
| Total | 239 (100.0) |  | 94.8 | 5.2 |  |  | 67.5 | 32.5 |  |  | 100.0 | 0.0 |  |  | 98.7 | 1.3 |  |  | 64.3 | 35.7 | |  |
| Age (years) |  |  |  |  |  |  |  |  |  |  |  |  |  |  |  |  |  |  |  |  | |  |
| Median (range) | 68 (61-76) |  | 69 (61-76) | 66.5 (59-71.8) | 0.720 |  | 68 (60-75) | 68.5 (62-76) | 0.539 |  | 68 (61-76) | - | - |  | 68 (61-75.8) | 66 (63-72.5) | 0.907 | | 67 (62-75) | 69 (58.8-76.3) | | 0.787 |
|  |  |  |  |  |  |  |  |  |  |  |  |  |  |  |  |  |  |  |  |  | |  |
| Sex |  |  |  |  |  |  |  |  |  |  |  |  |  |  |  |  |  |  |  |  | |  |
| Men | 56.5 |  | 57.9 | 33.3 | 0.094 |  | 54.4 | 59.2 | 0.490 |  | 56.2 | 0.0 | - |  | 56.8 | 66.7 | 0.731 | | 59.9 | 49.4 | | 0.132 |
| Women | 43.5 |  | 42.1 | 66.7 |  |  | 45.6 | 40.8 |  |  | 43.8 |  |  |  | 43.2 | 33.3 |  |  | 40.1 | 50.6 | |  |
|  |  |  |  |  |  |  |  |  |  |  |  |  |  |  |  |  |  |  |  |  | |  |
| Type of Monoclonal protein |  |  |  |  |  |  |  |  |  |  |  |  |  |  |  |  |  |  |  |  | |  |
| IgG | 53.1 |  | 54.3 | 41.7 | 0.488 |  | 56.5 | 51.4 | 0.626 |  | 52.8 | 0.0 | - |  | 53.7 | 33.3 | <0.001 | | 53.5 | 49.4 | | 0.839 |
| IgA | 27.2 |  | 25.8 | 41.7 |  |  | 27.9 | 26.4 |  |  | 27.2 | 0.0 |  |  | 27.1 | 0.0 |  |  | 26.1 | 30.4 | |  |
| Light chains | 13.4 |  | 13.1 | 16.7 |  |  | 11.4 | 17.1 |  |  | 13.6 | 0.0 |  |  | 13.6 | 0.0 |  |  | 12.6 | 15.2 | |  |
| Others | 6.3 |  | 6.8 | 0.0 |  |  | 7.0 | 5.2 |  |  | 6.4 | 0.0 |  |  | 5.7 | 66.7 |  |  | 7.7 | 5.1 | |  |
|  |  |  |  |  |  |  |  |  |  |  |  |  |  |  |  |  |  |  |  |  | |  |
| History of MGUS | 8.1 |  | 7.3 | 16.7 | 0.481 |  | 7.1 | 10.5 | 0.671 |  | 8.2 | 0.0 | - |  | 8.0 | 0.0 | 0.865 | | 9.2 | 1.3 | | 0.079 |
|  |  |  |  |  |  |  |  |  |  |  |  |  |  |  |  |  |  |  |  |  | |  |
| ISS stage |  |  |  |  |  |  |  |  |  |  |  |  |  |  |  |  |  |  |  |  | |  |
| I/II | 70.4 | | 70.2 | 70.0 | 0.989 |  | 69.5 | 74.6 | 0.448 |  | 71.5 | 0.0 | - |  | 70.4 | 0.0 | 0.125 | | 70.9 | 69.0 | | 0.780 |
| III | 29.6 |  | 29.8 | 30.0 |  |  | 30.5 | 25.4 |  |  | 28.5 |  |  |  | 29.6 | 100.0 |  |  | 29.1 | 31.0 | |  |
|  |  |  |  |  |  |  |  |  |  |  |  |  |  |  |  |  |  |  |  |  | |  |
| Cytogenetic profile (N=166) |  |  |  |  |  |  |  |  |  |  |  |  |  |  |  |  |  |  |  |  | |  |
| High-risk cytogenetic by FISH* | 8.6 |  | 9.4 | 0.0 | 0.396 |  | 6.5 | 11.1 | 0.343 |  | 8.7 | 0.0 | - |  | 9.0 | 0.0 | - |  | 10.1 | 4.1 | | 0.215 |
| Standard risk | 91.4 |  | 90.6 | 100.0 |  |  | 93.5 | 88.9 |  |  | 91.3 |  |  |  | 91.0 | - |  |  | 89.9 | 95.9 | |  |
|  |  |  |  |  |  |  |  |  |  |  |  |  |  |  |  |  |  |  |  |  | |  |
| LDH |  |  |  |  |  |  |  |  |  |  |  |  |  |  |  |  |  |  |  |  | |  |
| High | 8.5 |  | 8.4 | 12.5 | 0.690 |  | 5.6 | 14.3 | 0.060 |  | 8.6 | 0.0 | - |  | 8.9 | 0.0 | 0.588 | | 8.2 | 9.3 | | 0.823 |
| Normal | 91.5 |  | 91.6 | 87.5 |  |  | 94.4 | 85.7 |  |  | 91.4 |  |  |  | 91.1 | 100.0 |  |  | 91.8 | 90.7 | |  |
|  |  |  |  |  |  |  |  |  |  |  |  |  |  |  |  |  |  |  |  |  | |  |
| Kidney failureꝉ |  |  |  |  |  |  |  |  |  |  |  |  |  |  |  |  |  |  |  |  | |  |
| No | 77.6 |  | 77.3 | 75.0 | 0.857 |  | 81.2 | 71.4 | 0.102 |  | 78.1 | 0.0 | - |  | 76.6 | 100.0 | 0.339 | | 79.3 | 78.7 | | 0.919 |
| Yes | 22.4 |  | 22.7 | 25.0 |  |  | 18.8 | 28.6 |  |  | 21.9 |  |  |  | 23.4 | 0.0 |  |  | 20.7 | 21.3 | |  |
|  |  |  |  |  |  |  |  |  |  |  |  |  |  |  |  |  |  |  |  |  | |  |
| PBSCT |  |  |  |  |  |  |  |  |  |  |  |  |  |  |  |  |  |  |  |  | |  |
| Yes | 40.9 | | 41.0 | 41.7 | 0.964 |  | 43.6 | 36.5 | 0.307 |  | 40.7 | 0.0 | - |  | 41.3 | 33.3 | 0.780 | | 42.0 | 39.2 | | 0.688 |
| No | 59.1 |  | 59.0 | 58.3 |  |  | 56.4 | 63.5 |  |  | 59.3 |  |  |  | 58.7 | 66.7 |  |  | 58.0 | 60.8 | |  |

^a^ Except where specified.

MGUS: monoclonal gammopathy of undetermined significance. LDH: lactate dehydrogenase. PBSCT: Peripheral blood stem cell transplantation

*defined by detection of del(17p), t(4;14) or t(14;16). **^ꝉ^**Kidney failure defined as creatinine ≥ 2 mg/d

**Table S2.** Patients’ characteristics according to the CTLA4 rs2311775 and CTLA4 rs733618 genotypes.

|  | **Total** |  | **CTLA4 rs2311775** | | |  | **CTLA4 rs733618** | | |
| --- | --- | --- | --- | --- | --- | --- | --- | --- | --- |
|  | ***%a*** |  | **AA+AG** | **GG** | ***P*** |  | **CC+CT** | **TT** | ***P*** |
| Total | 239 (100.0) |  | 90.5 | 9.5 |  |  | 7.5 | 92.5 |  |
| Age (years) |  |  |  |  |  |  |  |  |  |
| Median (range) | 68 (61-76) |  | 68.0 (61-75.5) | 70.0 (64-75.3) | 0.480 |  | 68.0 (61-73) | 69.0 (61.5-76) | 0.426 |
|  |  |  |  |  |  |  |  |  |  |
| Sex |  |  |  |  |  |  |  |  |  |
| Men | 56.5 |  | 55.2 | 54.5 | 0.950 |  | 50.0 | 57.0 | 0.564 |
| Women | 43.5 |  | 44.8 | 45.5 |  |  | 50.0 | 43.0 |  |
|  |  |  |  |  |  |  |  |  |  |
| Age groups |  |  |  |  |  |  |  |  |  |
| ≤ 69 years | 53.0 |  | 54.1 | 45.5 | 0.439 |  | 64.7 | 52.1 | 0.314 |
| > 69 years | 47.0 |  | 45.9 | 54.5 |  |  | 35.3 | 47.9 |  |
|  |  |  |  |  |  |  |  |  |  |
| Type of Monoclonal protein |  |  |  |  |  |  |  |  |  |
| IgG | 53.1 |  | 54.8 | 45.5 | 0.284 |  | 66.7 | 52.0 | 0.678 |
| IgA | 27.2 |  | 26.7 | 22.8 |  |  | 16.7 | 28.0 |  |
| Light chains | 13.4 |  | 12.4 | 22.7 |  |  | 11.1 | 13.6 |  |
| Others | 6.3 |  | 6.2 | 9.0 |  |  | 5.6 | 6.3 |  |
|  |  |  |  |  |  |  |  |  |  |
|  |  |  |  |  |  |  |  |  |  |
| History of MGUS | 8.1 |  | 8.7 | 4.8 | 0.701 |  | 11.1 | 7.8 | 0.206 |
|  |  |  |  |  |  |  |  |  |  |
| ISS stage |  |  |  |  |  |  |  |  |  |
| I/II | 70.4 |  | 70.9 | 64.7 | 0.590 |  | 53.3 | 71.8 | 0.131 |
| III | 29.6 |  | 29.1 | 35.3 |  |  | 46.7 | 28.2 |  |
|  |  |  |  |  |  |  |  |  |  |
| Cytogenetic profile |  |  |  |  |  |  |  |  |  |
| High-risk cytogenetic by FISH* | 8.6 |  | 9.6 | 0.0 | 0.282 |  | 0.0 | 9.3 | 0.290 |
| Standard risk | 91.4 |  | 90.4 | 100.0 |  |  | 100.0 | 90.7 |  |
|  |  |  |  |  |  |  |  |  |  |
| LDH |  |  |  |  |  |  |  |  |  |
| High | 8.5 |  | 8.7 | 8.3 | 0.968 |  | 0.0 | 9.2 | 0.253 |
| Normal | 91.5 |  | 91.3 | 91.7 |  |  | 100.0 | 90.8 |  |
|  |  |  |  |  |  |  |  |  |  |
| Kidney failure |  |  |  |  |  |  |  |  |  |
| No | 77.6 |  | 78.3 | 70.0 | 0.394 |  | 61.1 | 79.0 | 0.080 |
| Yes | 22.4 |  | 21.7 | 30.0 |  |  | 38.9 | 21.0 |  |
|  |  |  |  |  |  |  |  |  |  |
| PBPCs |  |  |  |  |  |  |  |  |  |
| Yes | 40.9 |  | 41.1 | 38.1 | 0.792 |  | 52.9 | 39.9 | 0.292 |
| No | 59.1 |  | 58.9 | 61.9 |  |  | 47.1 | 60.1 |  |

^a^ Except where specified.

MGUS: monoclonal gammopathy of undetermined significance. LDH: lactate dehydrogenase. PBSCT: Peripheral blood stem cell transplantation

*defined by detection of del(17p), t(4;14) or t(14;16). **^ꝉ^**Kidney failure defined as creatinine ≥ 2 mg/d
